# First-line combination therapy of immunotherapy plus anti-angiogenic drug for thoracic SMARCA4-deficient undifferentiated tumors in AIDS: a case report and review of the literature

**DOI:** 10.3389/fimmu.2024.1473578

**Published:** 2025-01-09

**Authors:** Xiaoling Wei, Xiangju Xing, Wei Yao, Changzheng Wang, Yajie Xiao, Xianzhi Du

**Affiliations:** ^1^ Department of Respiratory Medicine, The Third Affiliated Hospital of Chongqing Medical University, Chongqing, China; ^2^ Department of Translational Medicine, Shenzhen Yucebio Technology Co., Ltd., Shenzhen, China; ^3^ Department of Respiratory Medicine, The Second Affiliated Hospital of Chongqing Medical University, Chongqing, China

**Keywords:** thoracic SMARCA4-deficient undifferentiated tumor, aids, immunotherapy, anti-angiogenic drug, lung cancer

## Abstract

**Background:**

Thoracic SMARCA4-deficient undifferentiated tumors (SMARCA4-UT) exhibit a notably aggressive phenotype, which is associated with poor patient survival outcomes. These tumors are generally resistant to conventional cytotoxic chemotherapy, thereby limiting the availability of effective treatment options.

**Case presentation:**

We describe a 69-year-old AIDS patient who initially presented with a fused, enlarged lymph node on the right clavicle and mild, unexplained pain under the right axilla that worsened with severe coughing episodes. An initial chest CT scan revealed multiple nodular and mass shadows in the mediastinum and multiple nodules in both lungs, as well as a small amount of pericardial effusion. Additionally, serum biomarkers of lung cancer were abnormal as follows: carcinoembryonic antigen (CEA) at 13.74 ng/mL, cytokeratin 19 fragment (CYFRA21-1) at 6.82 ng/mL, neuron-specific enolase (NSE) at 25.49 ng/mL, and progastrin-releasing peptide precursor (ProGRP) at 89.35 pg/mL. Subsequent pathology confirmed SMARCA4-deficient undifferentiated tumors. Considering that the weak immune status and intermediate PD-L1 level, the patient was treated with a first-line combination therapy of immunotherapy and anti-angiogenic drug instead of chemo-immunotherapy. The patient responded well to immunotherapy combining anti-angiogenic drugs and achieved an overall survival for more than 22 months.

**Conclusion:**

Our study presented a rare case of thoracic SMARCA4-deficient undifferentiated tumors and AIDS, suggesting that first-line immunotherapy plus anti-angiogenic drugs as a potential therapeutic option for SMARCA4-UT patients under specific conditions.

## 1 Introduction

The *SMARCA4* gene, located on the p arm of the 19th chromosome, is involved in encoding the BRG1 protein. This protein functions as one of the two exclusive catalytic subunits of the switch/sucrose-nonfermenting (SWI/SNF) complex, a chromatin-remodeling protein complex. The counterpart of BRG1 is the BRM protein, which is encoded by the SMARCA2 gene (1). The SWI/SNF complex, which consists of several proteins including the well-documented INI-1 from the *SMARCB1* gene, plays a crucial role as a tumor suppressor by modulating transcription and promoting cell differentiation (2–4). Loss of function of the SMARCA4 gene has been associated with the development of several high-grade tumors, including those of originating in the endometrium, gastrointestinal tract, lung, ovary, and central nervous system (5–9). Morphologically, tumors with inactivated SMARCA4 display a diverse range of phenotypes. These include both differentiated forms, such as adenocarcinomas and squamous cell carcinomas, as well as undifferentiated tumors characterized by a spectrum of rhabdoid features. This diversity underscores the complex and critical role that the SMARCA4 gene plays in maintaining cellular integrity and preventing the onset of malignancy.

Thoracic SMARCA4-deficient undifferentiated tumors represent a distinct entity from SMARCA4-deficient non-small cell lung carcinoma (NSCLC). They are predominantly found in young to middle-aged adults, with a marked male preponderance and a significant history of tobacco use (10, 11). These tumors are characterized by expansile growth and typically arise in the lung, mediastinum, hilum, or pleura, and are often associated with metastatic dissemination. It is crucial to conduct a comprehensive clinical evaluation, as similar tumors can originate in other regions, such as the abdomen, and then metastasize to the thoracic area. These thoracic SMARCA4-deficient tumors are highly aggressive, portending a poor prognosis, with reported median overall survival for patients is reported to be between 4 to 7 months (12, 13). Their resistance to conventional cytotoxic chemotherapy underscores the urgent need for alternative and effective management strategies. Given the rarity and complexity of these tumors, a multidisciplinary approach to diagnosis and treatment is essential, with an emphasis on novel therapeutic options that may improve patient outcomes.

In this study, we present a rare and intriguing patient with thoracic SMARCA4-deficient undifferentiated tumors (SMARCA4-UT) who also suffered from acquired immune deficiency syndrome (AIDS). This patient achieved a successful treatment outcome with a combination of immunotherapy and an anti-angiogenic drug, underscoring the promise of this therapeutic approach. Furthermore, we conducted an extensive review of the literature, including all reported cohorts and individual cases, to provide a comprehensive overview of the current treatment strategies for thoracic SMARCA4-deficient undifferentiated tumors.

## 2 Case presentation

In October 2021, a 69-year-old male patient was admitted to our hospital with a primary complaint of unexplained mild pain under the right armpit, which significantly worsened during severe coughing episodes. Upon thorough inquiry, it emerged that the patient had a substantial history of smoking, averaging 20 cigarettes daily for 30 years, and a parallel history of alcohol consumption, with an estimated intake of 50ml daily. Furthermore, the patient’s medical history included an HIV diagnosis in 2015, following which he was initiated on a daily regimen of oral antiretroviral therapy. This regimen comprised efavirenz 600mg, lamivudine 0.3g, and tenofovir disoproxil fumarate 300mg, and was maintained continuously for six years. Although the exact viral load at the time of diagnosis and in subsequent tests was not recorded, the patient reported well-managed AIDS throughout his treatment course. Also, regular follow-up assessments revealed an undetectable HIV viral load, indicating effective control of the infection.

Upon the patient’s admission, a thorough physical examination was conducted, which identified an enlarged lymph node, approximately 2 cm in diameter, located above the right clavicle. A computed tomography (CT) scan was promptly performed, revealing multiple nodular and mass shadows within the mediastinum, numerous nodular shadows and fibrotic lesions in both lungs, a small amount of pericardial effusion, and calcification in the aorta and coronary arteries. Following these findings, endobronchial ultrasound-guided mediastinal lymph node biopsies were performed. The cytological analysis of the aspirated cells suggested the presence of malignant cells, although the specific type and origin remained indeterminate.

**Figure 1:**
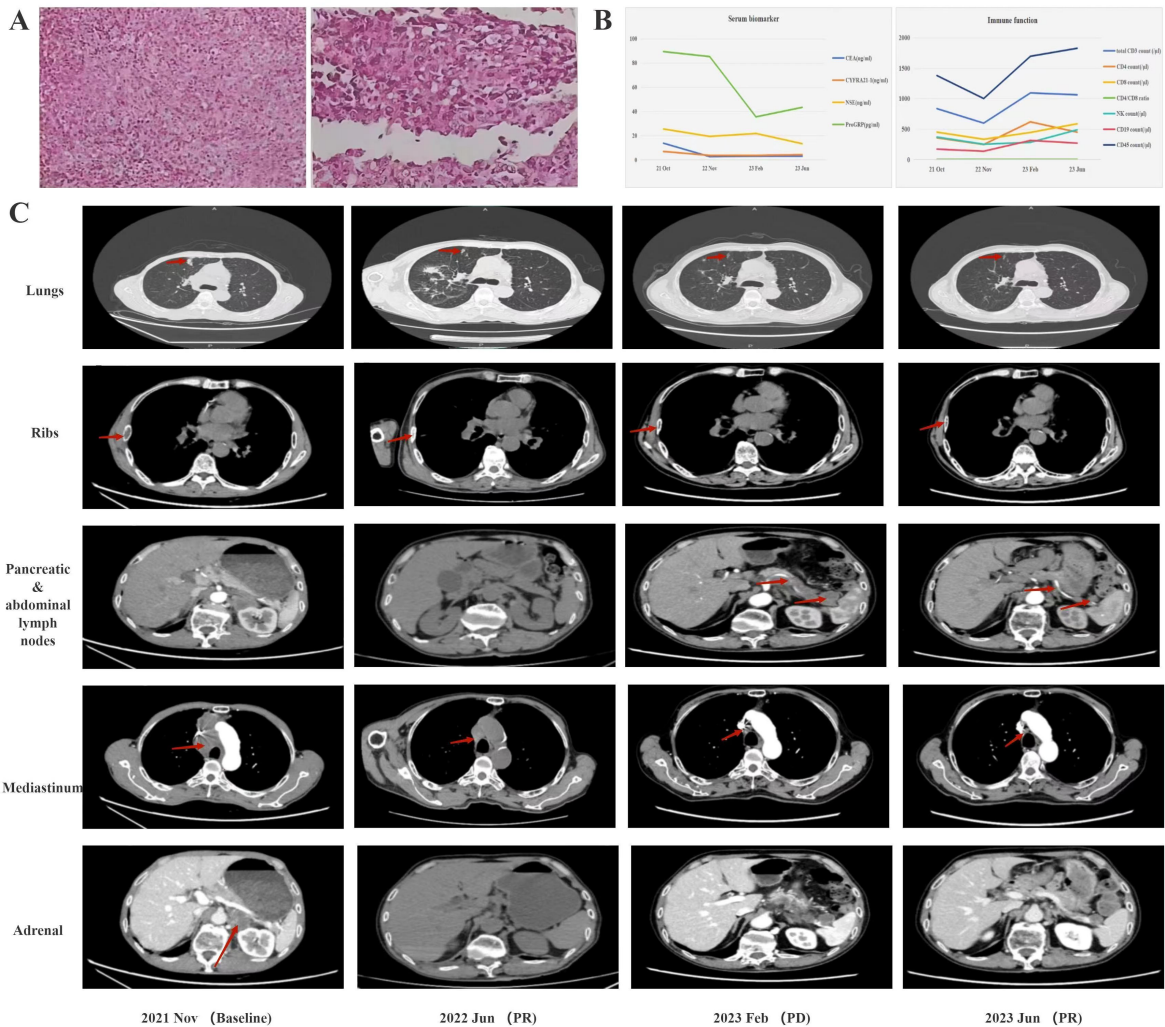
**(A)** Pathological findings at diagnosis. **(B)** serum biomarkers during treatment. **(C)** CT findings during treatment computed tomography.

To achieve a definite diagnosis, the right supraclavicular lymph node was surgically removed. The results of the immunohistochemistry (IHC) analysis showed the following tumor cell markers: EMA(+), CD3(-), CD5(-), CD10(-), CD19(-), CD20(-), CD21(-), CD30(-), CD34(+), CD56(-), CD68(-), CD117(-), P40(-), P63(-), CK7(-), NapsinA(-), TTF-1(sporadically+), CgA(-), Ki-67(80-90%), Mum-1(-), C-myc(-), BCL-2(-), BCL-6(-), S-100(-), INI-1(+), Villin(-), CDX2(-), NUT(+), SYN(focally+), CK(focally+), P53(strongly+),3 5BH11(focally+), CAM5.2(sporadically+), EBER-ISH(-), Vimentin(+) and SMARCA4 (-) (shown in **Figure 1A**). Moreover, serum biomarker examination for lung cancer were abnormal as follows: carcinoembryonic antigen (CEA) at 13.74 ng/mL, cytokeratin 19 fragment (CYFRA21-1) at 6.82 ng/mL, neuron-specific enolase (NSE) at 25.49 ng/mL, and progastrin-releasing peptide precursor (ProGRP) at 89.35 pg/mL (shown in **Figure 1C**). Immune function test yielded as follows: total CD3 count at 835cell/μL, CD4 count at 356cell/μL, CD8 count at 449cell/μL, CD4/CD8 ratio at 1.26, NK count at 369cell/μL, CD19 count at 170cell/μL, CD45 count at 1378cell/μL. Subsequent enhanced CT scans of the chest and abdomen revealed multiple metastases in the mediastinum, supraclavicular lymph nodes, ribs, left adrenal gland, and the lung. Accordingly, he was diagnosed as stage IV thoracic SMARCA4-deficient undifferentiated carcinoma.

The next-generation sequencing results did not show any significant gene mutations, and PD-L1 expression was positive at 10%. Given the patient’s compromised immune status and moderate PD-L1 expression, he was treated with sintilimab 200mg and bevacizumab 300mg (every 3 or 4 weeks per cycle) in November 2021 and partial remission achieved soon in June 2022. Throughout the follow-up period, the patient exhibited a favorable response to treatment, with serum biomarkers returning to normal levels and a marked improvement in immune function (**Figure 1B**). However, a follow-up CT scan in February 2023 revealed enlarged lymph nodes in the pancreas and abdominal cavity (**Figure 1C**). Consequently, the patient was transferred to sintilimab 200mg (every 3 or 4 weeks per cycle) and anlotinib 12mg (daily with a 2-week on/1-week off regimen per cycle) till he died for suspected cardiovascular sequelae of the COVID-19 in September 2023. It is noteworthy that the patient derived considerable benefit from the combination of immunotherapy and anti-angiogenic drugs, achieving an overall survival (OS) of more than 22 months.

## 3 Discussion

Patients with thoracic SMARCA4-deficient undifferentiated tumors are confronted with an exceedingly poor prognosis, with a median overall survival that typically ranges from only 4 to 7 months. Given this short survival span, the urgent development of effective treatment strategies is of paramount importance (12, 13). Here, we presented a rare case with thoracic SMARCA4-deficient undifferentiated tumors who also had HIV. This patient was successfully treated with a first-line regimen of immunotherapy combined with an anti-angiogenic drug, and he benefited from this treatment for more than 22 months. This case highlights the potential of immunotherapy and anti-angiogenic drugs as a viable treatment option for thoracic SMARCA4-deficient undifferentiated tumors, particularly in patients with comorbid conditions such as AIDS.

The advent of antiretroviral therapy (ART) has significantly enhanced the life expectancy of individuals infected with the human immunodeficiency virus. However, despite this medical breakthrough, there has been a disconcerting rise in cancer-related mortality among patients with HIV or AIDS, with lung cancer emerging as a particularly concerning trend (14). For these patients, treatments like chemotherapy and radiation therapy are known to suppress the immune response and pose the risk of causing a temporary yet significant drop in CD4+ T-cell counts. This reduction in key immune cells is not only indicative of a compromised immune system but is also directly associated with a heightened risk of mortality (15). Laboratory investigations have revealed that memory CD4+ T-cells in HIV-infected individuals frequently have an elevated level of the PD-1 protein. This finding implies that immune checkpoint inhibitors (ICIs) may offer a promising approach to rejuvenate the depleted CD4+ T-cell population in these patients. The strategic blockade of the PD-1 and PD-L1 interaction has demonstrated the capacity to disrupt the latent state of HIV within cells of individuals on ART, prompting an increase in viral replication. Importantly, this intervention also stimulates the reactivation of key immune functions within CD8+ and CD4+ T-cells, encompassing cytokine and cytotoxic agent production, as well as cellular proliferation (16). Encouragingly, recent real-world observational studies along with targeted research have begun to delineate the potential of immunotherapy as a feasible and effective therapeutic approach for HIV-positive/AIDS patients afflicted with solid tumors. This is especially true when their HIV infection is skillfully managed and maintained under effective control (17–21).

Additionally, we conducted a comprehensive literature review on SMARCA4-UT treating with immunotherapy. Currently, marketing ICIs that target PD-1/PD-L1 include pembrolizumab, nivolumab, cemiplimab, atezolizumab, avelumab, and durvalumab, which are either approved for clinical use or under clinical investigation in various cancer types. The majority of the published studies have focused on establishing a definitive diagnosis of this particular subtype through the use of sophisticated diagnostic techniques. There has also been a strong emphasis on the study of primary lesions arising from unusual anatomical sites (22–26). However, clinical evidence regarding the efficacy of ICI treatments for SMARCA4-UT remains scarce (**Table 1**).

**Table 1:** Current clinical evidences for immunotherapy in thoracic SMARCA4-deficient undifferentiated tumors (SMARCA4-UT) patients.

Reference	No of patients	Treatment	Response
Lin et al. (27)	25	ICI+chemotherapy	PFS 26.8 months ORR 71.4%
Wang et al. (28)	16	PD-1-inhibitor-based therapy	median OS not yet reached
Zhou et al. (29)	5	immunotherapy	ORR 80% OS 10.7- 33.6 months
Justine et al. (30)	9	nivolumab + ipilimumab	OS 2-6 months for 8 patients with “immune desert” phenotype OS 24 months for 1 patient with “immune-enriched” phenotype
Luo et al. (31)	8	immunotherapy	ORR 50%
Kawachi et al. (32)	2	atezolizumab + bevacizumab + chemotherapy	PFS 2-17 months
Al-Shbool et al. (33)	2	pembrolizumab+chemotherapy	1 patient not respond PFS (1 patient) 15 months
Shi et al. (34)	2	tislelizumab;atezolizumab +bevacizumab	1 patient (atezolizumab +bevacizumab)not respond PFS(tislelizumab) 6 months
Mashell et al. (35)	1	pembrolizumab + chemotherapy + radiotherapy	55% reduction in the size of the primary tumor
Yang et al. (36)	1	tislelizumab + chemotherapy	PFS 10 months
Yoshio Nakano et al. (37)	1	nivolumab + ipilimumab	OS 4 months
Takahiro et al. (38)	1	atezolizumab+ chemotherapy	PFS 7 months
Li et al. (39)	1	camrelizumab + chemotherapy	PFS 9 months
Dong et al. (40)	1	tislelizumab + chemotherapy	PFS 9 months
Nambirajan et al. (41)	1	pembrolizumab + ipilimumab	PFS 22 months
Takada et al. (42)	1	pembrolizumab	PFS 8 months
Henon et al. (43)	1	pembrolizumab	PFS 11 months
Anžič et al. (44)	1	pembrolizumab followed by pembrolizumab + ipilimumab	PFS 12 months
Iijima et al. (45)	1	nivolumab	PFS 22 months
Hanona et al. (46)	1	nivolumab	discontinued for weakness after 2 cycles
Xiong et al. (47)	1	neo-adjuvant chemo-immunotherapy	complete pathological response
Kunimasa et al. (48)	1	neoadjuvant atezolizumab + bevacizumab	major pathological response
Our case	1	sintilimab + anti-angiogenic drugs	OS 22 months

PFS, progression-free survival; OS, overall survival; ORR, overall response rate.

### 3.1 Clinical studies for adjuvant single-agent or combination immunotherapy in SMARCA4-UT patients

Lin et al. evaluated the use of ICI in combination with chemotherapy as a first-line treatment in patients with stage IV SMARCA4-UT (n=25), and showed that the median progression-free survival (PFS) was significantly improved compared to conventional chemotherapy alone (26.8 months versus 2.73 months, p=0.0437) (27). Wang et al. conducted a clinical study with small sample size and showed that the median OS for the PD-1 inhibitor group had not yet been reached at the time of the study analysis, while those who underwent chemotherapy treatment (n=9) have a median OS of 14.9 months (p= 0.105) (28). Zhou et al. evaluated 5 cases treated with immunotherapy and found an objective response rate of 80% and OS ranging from 10.7 to 33.6 months (29). Justine et al. performed an observational study in 9 patients to investigate the correlation between the tumor microenvironment and the clinical response. Among them, 8 patients with an “immune desert” phenotype did not respond to immunotherapy, while 1 patient with an immune-enriched tumor microenvironment had a rapid and durable partial response for 24 months (30). Lin et al. showed that SMARCA4-UT patients had significantly worse progression time and OS than SMARCA4-NSCLC patients. Besides, there was also a 50% ORR in 8 patients who received immunotherapy (31).

### 3.2 Case series for adjuvant single-agent or combination immunotherapy in SMARCA4-UT patients

Kawachi et al. reported 2 cases treated with a first-line therapy combination of atezolizumab, bevacizumab, paclitaxel, and carboplatin, which resulted in a PFS ranging from 10 to 17 months (32). IAl-Shbool et al. described the immunotherapy treatment experience of two Western patients (33). One patient did not respond and unfortunately passed away shortly thereafter, but the other patient exhibited good tolerance to the pembrolizumab plus chemotherapy regimen for 15 months. Shi et al. presented a 50-year-old male patient experiencing progressively worsening respiratory failure who responded remarkably to tislelizumab monotherapy over the course of 6 treatment cycles (34). However, other patients in this study did not respond to pembrolizumab monotherapy or the combination of atezolizumab plus bevacizumab.

### 3.3 Case reports for adjuvant combination immunotherapy in SMARCA4-UT patients

Mashell et al. presented a 62-year-old female patient who underwent combination therapy with pembrolizumab + carboplatin+etoposide+ radiotherapy and achieved a 55% reduction in the size of the primary tumor (35). Yang et al. documented a 51-year-old male patient who achieved a disease-free survival of more than 10 months with a second-line regimen comprising tislelizumab plus etoposide+carboplatin (36). Yoshio Nakano et al. reported a 73-year-old male patient who exhibited a significant reduction in both the primary tumor and metastatic lesions after only two cycles of ipilimumab and nivolumab (37). Takahiro et al. described a 72-year-old male patient with dyspnea and hemoptysis who achieved a stable disease response to atezolizumab in combination with carboplatin and nab-paclitaxel for 7 months (38). Li et al. reported a 63-year-old male patient treated with chemoinfusion along with camrelizumab, who obtained a durable partial response for 9 months (39). Dong et al. published a 56-year-old male patient who was treated with tislelizumab plus etoposide and cisplatin for 9 months without tumor progression (40). Nambirajan et al. reported a 41-year-old male patient with a partial response to pembrolizumab plus ipilimumab for 22 months (41).

### 3.4 Case reports for adjuvant single-agent immunotherapy in SMARCA4-UT patients

Takada et al. presented a 70-year-old female patient who received a first-line pembrolizumab monotherapy and achieved a durable partial response for at least 8 cycles (42). Henon et al. reported a 58-year-old female case who had a significant treatment response to pembrolizumab for 11 months, irrespective of PD-L1 expression levels (43). Anžič et al. showed a 41-year-old male SMARCA4-UT case who experienced progression in cervical and mediastinal lymph nodes after completing eight cycles of pembrolizumab monotherapy. However, upon switching the treatment regimen to a combination of pembrolizumab and ipilimumab, the patient exhibited mixed responses after an additional four cycles (44). Iijima et al. documented a 76-year-old male patient receiving a third-line nivolumab monotherapy and achieved a nearly complete response for 22 months (45). Hanona et al. presented a 40-year-old man with SMARCA4-UT in pleura, who was treated with a second-line nivolumab monotherapy but had to discontinue treatment after 2 cycles due to weakness (46).

### 3.5 Case reports for neo-adjuvant combination immunotherapy in advanced SMARCA4-UT patients

Xiong et al. described a 64-year-old male case who received neo-adjuvant chemo-immunotherapy and achieved a complete pathological response (47). Kunimasa et al. presented a 56-year-old man treated with a neoadjuvant therapy consisting of atezolizumab plus bevacizumab, with no recurrence observed during a follow-up period of 9 months (48).

To some extent, chemo-immunotherapy has already shown a potential efficacy in SMARCA4-UT. In addition, targeted therapy has been shown to be effective in achieving remarkable complete responses in patients with SMARCA4-UT harboring actionable mutations, such as anaplastic lymphoma kinase (ALK) rearrangements, in both first- and second-line settings (31, 49). However, the exploration of other combination treatments, such as the use of anti-angiogenic drugs and histone deacetylase inhibitors, may also yield beneficial outcomes for patients with SMARCA4-UT. These diverse therapeutic approaches may provide additional avenues for the management of this aggressive disease.

In this study, the patient underwent genetic testing, which revealed no targetable gene mutations. Given the weak immune status and low PD-L1 level (10%), the patient was initially treated with sintilimab and bevacizumab initially. Throughout the follow-up period, the patient exhibited a favorable response to anti-cancer treatment and an improvement in immune function. However, a subsequent CT scan revealed enlarged lymph nodes in the pancreas and abdominal cavity. Considering the current clinical evidences showing the anlotinib can be an therapeutic option for those patients who previously treated with bevacizumab (50–52), the patient was transferred from sintilimab plus bevacizumab to sintilimab plus anlotinib. For example, Feng et al. showed that ICI plus anlotinib or apatinib resulted in a significantly improved median PFS than ICI plus bevacizumab in locally advanced or metastatic lung adenocarcinoma patients (3.3 months vs 1.2 months, P = 0.005) (51). Jiang et al. also demonstrated that anlotinib had a favorable activity and safety profile in advanced non-small cell lung cancer who were previously treated with bevacizumab (52).

Mechanically, the combination of anti-PD-1/PD-L1 antibodies with anti-angiogenic drugs has demonstrated a synergistic impact in cancer therapy (53). Anti-angiogenic drugs normalize the tumor vasculature, which enhances the immune cell infiltration and helps to transform “cold tumors” into “hot tumors”, thereby increasing the efficacy of immunotherapy (54, 55). These drugs promote the maturation of immune cells and facilitate their infiltration by inhibiting the interaction between VEGF and its receptor VEGFR2 on macrophages and T cells (56). This normalization process reduces hypoxia and decreases the secretion of VEGF, which in turn reduces the recruitment of immunosuppressive cells such as MDSCs and Tregs and lowers the expression of immune checkpoint molecules like PD-1, PD-L1, CTLA-4, and TIM-3 on these cells (57, 58). Additionally, vascular normalization also leads to more efficient priming of lymphocytes by antigen-presenting cells, polarization of tumor-associated macrophages (TAMs) towards an M1-like phenotype, and the accumulation of activated, IFN-γ-expressing CD8+ T cells around blood vessels (59, 60). M1-like TAMs are generally recognized as anti-tumor and pro-immunity macrophages. In the context of ICIs, these drugs are thought to act primarily on T cells, stimulating them to secrete IFN-γ, which in turn reduces endothelial VEGFA and increases the levels of CXCL-9, CXCL-10, and CXCL-11, contributing to tumor vascular normalization (61, 62). Consequently, anti-angiogenic drugs may improve the potency of immunotherapy by promoting vascular normalization.

Generally, our case indicates that first-line treatment with a combination of immunotherapy and anti-angiogenic drugs could be a promising therapeutic strategy for patients with SMARCA4-UT. This approach may be particularly pertinent for patients with specific conditions, such as those with AIDS, where the interplay between the tumor and the immune system is likely complicated by the pre-existing immune deficiency. Further studies will be conducted to more fully comprehend the therapeutic efficacy and the potential role of combining immunotherapy with anti-angiogenic drugs in the treatment of SMARCA4-UT.
